# Selective Labeling of Individual Neurons in Dense Cultured Networks With Nanoparticle-Enhanced Photoporation

**DOI:** 10.3389/fncel.2018.00080

**Published:** 2018-03-29

**Authors:** Ranhua Xiong, Peter Verstraelen, Jo Demeester, Andre G. Skirtach, Jean-Pierre Timmermans, Stefaan C. De Smedt, Winnok H. De Vos, Kevin Braeckmans

**Affiliations:** ^1^Laboratory of General Biochemistry and Physical Pharmacy, Faculty of Pharmaceutical Sciences, Ghent University, Ghent, Belgium; ^2^Centre for Nano- and Biophotonics, Faculty of Pharmaceutical Sciences, Ghent University, Ghent, Belgium; ^3^Laboratory of Cell Biology and Histology, Department of Veterinary Sciences, University of Antwerp, Antwerp, Belgium; ^4^Department of Molecular Biotechnology, Ghent University, Ghent, Belgium; ^5^College of Chemical Engineering, Jiangsu Key Lab of Biomass-Based Green Fuels and Chemicals, Nanjing Forestry University, Nanjing, China; ^6^Univ Lille 1, Univ Lille Nord France, IEMN, UMR 8520, Villeneuve D’Ascq, France; ^7^Univ Lille 1, Univ Lille Nord France, Lab Phys Lasers Atomes & Mol, UMR 8523, Villeneuve D’Ascq, France

**Keywords:** dendritic spine, gold nanoparticle, photoporation, primary neuronal culture, neuron labeling, SNAP

## Abstract

Neurodevelopmental and neurodegenerative disorders are characterized by subtle alterations in synaptic connections and perturbed neuronal network functionality. A hallmark of neuronal connectivity is the presence of dendritic spines, micron-sized protrusions of the dendritic shaft that compartmentalize single synapses to fine-tune synaptic strength. However, accurate quantification of spine density and morphology in mature neuronal networks is hampered by the lack of targeted labeling strategies. To resolve this, we have optimized a method to deliver cell-impermeable compounds into selected cells based on Spatially resolved NAnoparticle-enhanced Photoporation (SNAP). We show that SNAP enables efficient labeling of selected individual neurons and their spines in dense cultured networks without affecting short-term viability. We compare SNAP with widely used spine labeling techniques such as the application of lipophilic dyes and genetically encoded fluorescent markers. Using SNAP, we demonstrate a time-dependent increase in spine density in healthy cultures as well as a reduction in spine density after chemical mimicry of hypoxia. Since the sparse labeling procedure can be automated using an intelligent acquisition scheme, SNAP holds promise for high-content screening campaigns of neuronal connectivity in the context of neurodevelopmental and neurodegenerative disorders.

## Introduction

Many neurodevelopmental and neurodegenerative disorders are characterized by altered synaptic connectivity (Glausier and Lewis, 2013; Herms and Dorostkar, 2016). However, insufficient fundamental knowledge about the underlying mechanisms at the cellular and neuronal network levels precludes the development of disease-modifying treatments.

Primary neuronal cultures are frequently used *in vitro* models in studies of neuroplasticity due to their ability to spontaneously form synaptically connected networks (Cohen et al., 2008; Verstraelen et al., 2017). Synapses, in particular postsynaptic compartments known as dendritic spines, serve as sensitive morphological correlates of neuronal connectivity since they appear, disappear or display morphological modifications in response to learning paradigms (Segal, 2017). Spine motility, and hence the extent of electrical and biochemical compartmentalization, is mediated by the actin cytoskeleton (Lei et al., 2016). With a typical length between 0.5 μm and 4 μm (Papa et al., 1995), spines can be resolved using high-resolution confocal microscopy. However, accurate spine quantification is complicated due to the dense nature of mature neuronal networks with many dendrites and axons crossing each other, making a sparse labeling strategy imperative. Ideally, such a method allows selecting individual neurons that are sufficiently separated or have a particular phenotype of interest. To meet these requirements, we have optimized a recently reported technique called Spatially resolved NAnoparticle-enhanced Photoporation (SNAP; Xiong et al., 2017). This approach exploits the ability of plasmonic gold nanoparticles (AuNPs) to generate vapor nanobubbles (VNBs) upon pulsed laser irradiation (Skirtach et al., 2005; Xiong et al., 2016b). When a membrane-adsorbed AuNP receives a laser pulse, it rapidly heats up, causing the surrounding water to evaporate. This results in the formation of VNBs that expand and collapse, thereby creating transient pores in the plasma membrane allowing otherwise impermeable molecules to enter the cytoplasm. Spatial selectivity, after bulk incubation with AuNPs, is achieved by directing the laser beam to the cells of interest. In this study, we exploit the concept of SNAP for targeted delivery of the cell-impermeable and fluorescently labeled actin-binding dye phalloidin in individual primary neurons. This allows the visualization of dendritic spines with high contrast in dense neuronal networks. Using automated selection of Hoechst-labeled neuronal nuclei, we significantly increase the throughput, illustrating the potential of SNAP for high-content applications. We assess the toxicity and selectivity of SNAP and compare with a number of commonly used techniques for sparse labeling in terms of targeting specificity, speed and ease-of-use. As proof-of-concept, we quantify maturation-dependent and hypoxia-induced alterations in spine density.

## Materials and Methods

### Primary Hippocampal Cultures and Hypoxia Induction

This study was carried out in accordance with the recommendations of the ethical committee for animal experimentation of the University of Antwerp (approved ethical files 2014-53 and 2015-54).

Hippocampi were dissected from WT E18 C57Bl6 mouse embryos in HEPES (7 mM)-buffered Hanks Balanced Salt Solution (HBSS-HEPES), followed by trypsin digestion (0.05%; 10 min; 37°C) and mechanical dissociation. After centrifugation (5 min at 200 *g*), the cell pellet was resuspended in Minimal Essential Medium supplemented with 10% heat-inactivated normal horse serum and 30 mM glucose. Cells were plated in Poly-D-Lysin-coated 96-well plates (Greiner Cell coat, μClear), at 45,000 cells/cm^2^, and kept in a humidified CO_2_ incubator (37°C; 5% CO_2_). After 4 h, the medium was replaced with 150 μl B27-supplemented Neurobasal medium (NB-B27), containing Sodium Pyruvate (1 mM), Glutamax (2 mM) and glucose (30 mM). To suppress proliferation of non-neuronal cells, 1 μM arabinosylcytosine was added in 50 μl NB-B27 at the third day after plating. The cultures were grown without any further medium replacement until the time of analysis. Cell culture supplies were purchased from ThermoFisher.

For pharmacological induction of hypoxia, Dimethyloxalylglycine (DMOG; 10–100 μM; Sigma-Aldrich D3695) was added at 13 days *in vitro* (DIV) in 25 μl NB-B27, for 24 h. Controls received an equal volume of sterile water in NB-B27 (final volume of 25 μl).

### Spatially Resolved Nanoparticle-Enhanced Photoporation (SNAP)

At the indicated DIV, 130 μl NB-B27 was removed (~50 μl left) and cells were incubated at 37°C with cationic AuNPs of 70 nm (CU11-70-P30-50, Nanopartz Inc., Zeta-potential +30 mV) in 25 μl HBSS-HEPES at a final concentration of 1.8 × 10^8^ particles/ml. After a 30 min incubation period, the cell-permeant nuclear label Hoechst 33342 (final concentration 10 μg/ml in 25 μl HBSS-HEPES, ThermoFisher H3570) was added to the cells and incubated for another 15 min at room temperature. Subsequent imaging and photoporation were done at room temperature while the pH was maintained by the HEPES buffer. The Hoechst-stained cell culture was imaged using a confocal laser scanning microscope (Nikon C1si, 10× NA 0.45 CFI Apochromat, *λ*_ex_ 405 nm, *λ*_em_ 440 nm). A large overview image of 1.27 by 1.27 mm was obtained using the microscope’s image stitching feature. Nuclei were identified in the image as described before (De Vos et al., 2010) and a sparse selection of sufficiently separated cells was made with a custom-written Matlab script (available upon request). Afterwards, the local coordinates of the cells in the image were transformed to global coordinates of the microscope stage. Two minutes prior to the laser scanning treatment, 25 μl of AF488- or AF647-labeled phalloidin (final concentration 20 U/ml; ThermoFisher Scientific) in HBSS-HEPES was added to the cells. A homemade setup including a Nikon Ti epifluorescence microscope with programmable motorized stage and electronic timing system was used to generate and detect VNBs (Xiong et al., 2014). A pulsed laser (7 ns pulses at 20 Hz) was tuned at a wavelength of 561 nm (Opolette HE 355 LD, OPOTEK Inc.) and used for illumination of AuNPs to generate VNBs. A beam expander (#GBE05-A, Thorlabs) combined with iris diaphragm (#D37SZ, Thorlabs) was used to adjust the diameter of the laser beam to the size of a neuronal soma (~20 μm diameter). The laser pulse energy was monitored by an energy meter (J-25MB-HE&LE, Energy Max-USB/RS sensors, Coherent) synchronized with the pulsed laser. The laser fluence level was maintained at 2 J/cm^2^, i.e., well above the VNB threshold (~0.5 J/cm^2^ @ 561 nm and 7 ns pulse duration) of the 70 nm AuNP as previously determined (Xiong et al., 2014, 2016a).

### Confocal Imaging and Image Quantification

After photoporation, the sample was placed back in the incubator for at least 1 h to allow the cells to recover and permit equal phalloidin distribution throughout the targeted neuron. After gently washing excess phalloidin in the culture medium, labeled cells were imaged with a confocal laser scanning microscope using both low (10 × NA 0.45) and high (60 × W NA 1.20) magnification objective lenses (*λ*_ex_ = 488 nm, *λ*_em_ = 525 ± 25 nm or *λ*_ex_ = 647 nm, *λ*_em_ = 700 ± 40 nm). To visualize the entire neuronal network, the membrane-permeable dye SiR-tubulin (1 μM final concentration) was added for 30 min prior to imaging (*λ*_ex_ = 647 nm, *λ*_em_ = 705 ± 45 nm). Confocal images were usually acquired within 1 h after recovery from the photoporation procedure, but some cultures were imaged 24 h after SNAP. Quantification of dendritic spine density, i.e., the number of spines divided by the dendrite length, was done manually assisted by ImageJ software (Schneider et al., 2012).

### Determination of Cell Viability

The following approach was adopted to screen for potential cytotoxic effects of the photoporation procedure in 14 DIV cultures. First, the laser beam scanned a pre-defined square pattern covering most of the well (diagonal of the square = diameter of the well). Laser pulse frequency, laser beam diameter and stage speed were adjusted so that each location within the square received a single laser pulse. AuNP concentration, incubation times and laser fluence were identical to those used for image-guided SNAP. The following conditions were considered: −AuNP/−laser; +AuNP/−laser; −AuNP/+laser; +AuNP/+laser. Immediately after the photoporation procedure, the wells were replenished with conditioned medium and placed in an incubator at 37°C for 2 or 24 h. During the last hour, the cultures were incubated with 10 μl PrestoBlue reagent (ThermoFisher A13261), after which the fluorescence was measured in a plate reader (*λ*_ex_ 535 nm, *λ*_em_ 590 nm).

### Alternative Spine Labeling Strategies

Lipophilic dye labeling −14 DIV cultures were fixed (2% paraformaldehyde in 0.1 M phosphate buffer, 20 min at RT) and stained with the carbocyanine dye CM-DiI (1 μg/ml in PBS, 20 min, ThermoFisher C7001). At least 24 h passed between staining and imaging, allowing the hydrophobic dye to spread throughout the plasma membrane.

#### Mixed Culture

Hippocampi were dissected from WT and Black6.Cg-Tg(Thy1-YFP)16Jrs/J mice (The Jackson laboratory #003709) as described above. After obtaining the single cell suspension, the Thy1-YFP (YFP+) neurons were mixed with WT hippocampal cells at a 1:5 ratio. Cultures were grown in standard conditions until live imaging (14 DIV).

#### Sparse Transduction

At 12 DIV, primary neurons were incubated with CellLight Actin-RFP, BacMam 2.0 particles (MOI 15, ThermoFisher C10583). This transduction protocol led to the transduction of a few dozen neurons per well, with high expression levels at 14 DIV.

#### Photoconversion

To obtain Adeno-Associated Viral (AAV) particles encoding for an actin-binding photoconvertible protein, a mEos4b-LifeAct7 construct (Addgene #54813 deposited by Michael Davidson) was cloned into an AAV backbone with hSyn1 promoter (Addgene #51085 deposited by Jonathan Ting), followed by the generation of AAV particles using an AAV-DJ helper free packaging kit (Cell Biolabs VPK-400-DJ). Transduction at 0 DIV (0.5 μl crude lysate/well) resulted in the expression of the green (*λ*_ex_ 488 nm) mEos-LifeAct variant in the entire neuronal network. At 14 DIV, live cultures were placed on a confocal spinning disk microscope (UltraVIEW VoX, PerkinElmer) with photoconversion functionality (PhotoKinesis device, PerkinElmer). Every 2 min, a spot-shaped photoconversion pulse (405 nm, 100% laser power, 10 ms, diameter ~5 μm) was delivered in the middle of a neuronal cell body, after which images were acquired in the green (non-photoconverted; *λ*_ex_ 488 nm, *λ*_em_ 525 nm) and red channel (photoconverted; *λ*_ex_ 561 nm, *λ*_em_ 615 nm).

### Experimental Design and Statistical Analysis

For determination of cell viability with PrestoBlue, one isolation was considered with six wells per condition. For quantification of dendritic spine density, three independent isolations were considered. For each isolation, >1000 μm of dendrite stretches were analyzed on 10 selected neurons for each DIV or DMOG concentration.

Statistical analyses were carried out in SAS JMP Pro 12 software, and the detailed results of those tests are reported in Table 1. Shapiro-Wilk tests were used to check for normality. Since some of the data sets were not normally distributed, non-parametric tests were performed throughout the article. To assess the overall effect, Mann-Whitney (2 groups) or Kruskal-Wallis rank sums (>2 groups) tests were performed. When assessing the influence of culture age or DMOG treatment on spine density, these tests were performed within the method group (SNAP or DiI). For PrestoBlue measurements, these tests were performed within the time group (2 or 24 h). Conditional to the overall Kruskal-Wallis test, *post hoc* tests were performed. To detect differences across DIVs, Dunn all pairs tests for joint ranks were used as the non-parametric alternative for Bonferroni tests (Dunn, 1964). For the DMOG experiment, a Steel test for comparison with control was used as the non-parametric alternative for a Dunnett test (Steel, 1959). The data are represented as bar charts (mean + standard deviation).

**Table 1:** Detailed statistics for the executed experiments.

Figure	Description	Condition	Shapiro-Wilk	DF	Overall test	*Post hoc* test
2D	Cyototoxicity	2 h	*p* = 0.7953	3	KW *p* = 0.2064	NA
		24 h	*p* = 0.8160		KW *p* = 0.3755	NA
4B	DIVs	SNAP	*p* < 0.0001	2	KW *p* < 0.0001	Dunn all pairs—7–14 DIV *p* < 0.0001; 14–21 DIV *p* = 0.3657; 7–21 DIV *p* < 0.0001
		DiI	*p* = 0.3802	2	KW *p* = 0.002	Dunn all pairs—7–14 DIV *p* = 0.0324; 14–21 DIV *p* = 0.6017; 7–21 DIV *p* = 0.0024
4D	DMOG	SNAP	*p* = 0.0025	2	KW *p* = 0.0066	Steel with control—10 μM *p* = 0.0153; 100 μM *p* = 0.0157
		DiI	*p* = 0.0003	2	KW *p* = 0.0043	Steel with control—10 μM *p* = 0.0194; 100 μM *p* = 0.0048
**S1A**	Specificity		*P* = 0.2952	1	MW *p* = 0.009	NA
**S1B**	Spine morphology		*p* = 0.4426	4	KW *p* < 0.0001	Dunn all pairs—mEosLA-DiI *p* = 1; SNAP-DiI *p* = 1; SNAP-mEosLA *p* = 1; YFP-DiI *p* = 1; YFP-SNAP *p* = 1; YFP-mEosLA *p* = 0.6875; mEosLA-BacMam *p* = 0.0454; SNAP-BacMam *p* = 0.0071; DiI-BacMam *p* = 00064; YFP-BacMam *p* < 0.0001
**S2B**	YFP spines		*p* = 0.3817	1	MW *p* = 0.7494	NA
**S2C**	Functional integration	7 DIV	*p* = 0.0187	1	MW *p* = 0.9223	NA
		14 DIV	*p* = 0.1228	1	MW *p* = 0.5485	NA

*For all quantitative data, the p-values of Shapiro-Wilk tests for normality are listed, as are the degrees of freedom (DF) and p-values of the overall tests (KW: Kruskal-Wallis; MW: Mann-Whitney), and the nature and p-values of the post hoc tests*.

## Results

### SNAP Enables Fast and Targeted Labeling of Primary Hippocampal Neurons and Their Spines

**Figure 1:**
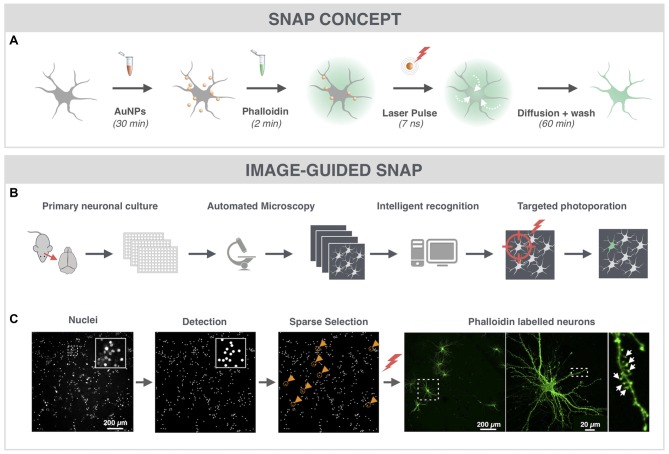
Spatially resolved NAnoparticle-enhanced Photoporation (SNAP) enables fast and targeted labeling of primary hippocampal neurons and their spines. **(A)** General workflow of SNAP. Intracellular delivery of phalloidin relies on the adsorption of cell-interactive plasmonic gold nanoparticles (AuNP) to the plasma membrane. Upon selective illumination of the cell of interest with 561 nm laser light, AuNPs heat up and induce vapor nanobubbles (VNBs). These VNBs transiently open the membrane, allowing the otherwise impermeable dye to enter the soma (white arrows). After washing and allowing time for intracellular diffusion of the dye to the neuronal extremities, completely labeled neurons can be monitored with high contrast. **(B)** Workflow of image-guided SNAP. Before the actual SNAP procedure is initiated, large field-of-view images are acquired of cells labeled with a cell-permeable marker. After image analysis and selection, the coordinates of the cells of interest are determined and fed into the SNAP setup for targeted photoporation. **(C)** Image-guided SNAP on primary neurons. Nuclei are stained with the membrane-permeable dye Hoechst. Using image analysis, all nuclei are first detected, followed by a selection of nuclei that are at least 200 μm apart (gold circles and arrowheads). The coordinates of the neurons of interest are used to guide the SNAP procedure. The high-resolution image of the phalloidin-labeled neuron shows dendritic spines (arrows) in high contrast without interference of fluorescence from overlapping dendrites or axons of nearby cells.

Since dendritic spines contain high actin concentrations we used the cell-impermeable fluorescently labeled F-actin dye phalloidin to label hippocampal neurons (Figure 1A). To quickly and automatically label a sparse set of neuronal cells within the dense network, we made use of image-guided SNAP (Figure 1B). This approach was based on the assumption that by automatically pre-selecting the target cells, we could maximize the distance between labeled the cells in the field of view. We also reasoned that by targeting the soma of neurons, we would maximize the labeling efficiency and cell selectivity. Therefore, cells were labeled with the membrane-permeable nuclear counterstain Hoechst, allowing facile detection of nuclei, which largely colocalize with the soma (Verschuuren et al., 2017). The positions of individual nuclei were identified by image processing and a random selection was made under the constraint that selected cells should be at least 200 μm apart (Figure 1C, gold circles and arrowheads). The coordinates of the selected cells were subsequently used for SNAP-assisted phalloidin labeling. Each cell received a single 7 ns laser pulse at the indicated position with a beam diameter of ~20 μm. As demonstrated in Figure 1C, only the targeted cells were successfully labeled. Since the photoporation beam was centered onto the nuclear area, phalloidin initially entered the soma of the cell, after which it diffused throughout the cell into the dendrites and labeled the dendritic spines (Figure 1A). The inset in Figure 1C shows that dendritic spines (arrows) could be unambiguously resolved. Although there is a theoretical possibility that neurites of non-targeted neurons, which are in close proximity to the target soma, may become transiently permeabilized as well, we did not observe such aspecific labeling, at least not with the acquisition settings that were suitable for imaging the target neuron. Therefore, we conclude that SNAP allows specific labeling of selected neurons with minimal off-target effects.

### SNAP-Assisted Phalloidin Labeling Is Selective and Non-Cytotoxic

**Figure 2:**
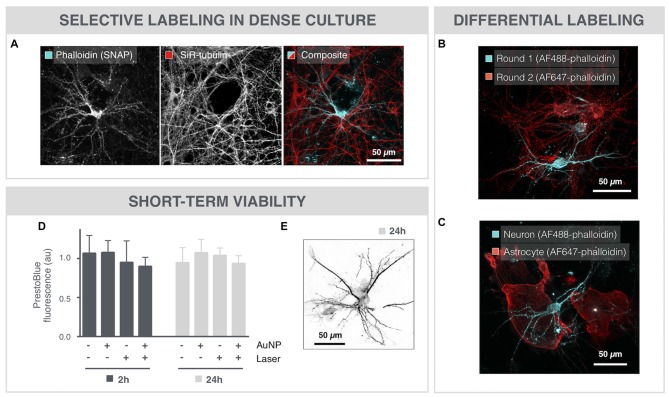
SNAP-assisted phalloidin labeling is selective and non-cytotoxic. **(A)** Microscopic image of an 18 DIV neuron that was selectively labeled with image-guided SNAP. The complete network is labeled with the membrane-permeable dye SiR-tubulin, whereas only the targeted neuron is labeled with phalloidin. **(B)** Repetitive SNAP procedure with two chromatic phalloidin variants (AF488, AF647) allows multicolor labeling of individual neurons in the same field of view.** (C)** The same procedure was used to selectively label astrocytes and neurons in different colors in the same culture. **(D)** Quantification of cell viability after 2 and 24 h shows no significant toxicity induced by the AuNPs or the 561 laser pulses, nor of the combination of both. **(E)** Microscopic image of a neuron, 24 h after SNAP-assisted phalloidin labeling.

Even in very dense cultures that were grown for 18 DIV, SNAP maintained target specificity allowing sparse labeling (Figure 2A). Furthermore, SNAP could be used to sequentially label individual neurons in the same field of view in different colors (Figure 2B). The same repetitive SNAP procedure could also be used to label different cell types in a single culture (Figure 2C).

To evaluate potential cytotoxicity induced by the SNAP procedure, cell viability was determined with PrestoBlue-based spectrophotometry after whole-field SNAP (Figure 2D). Neither incubation with AuNPs, nor illumination with the pulsed 561 nm laser elicited a significant cytotoxic effect after 2 or 24 h. Moreover, the combination of AuNPs and laser irradiation, and hence the formation of VNBs, did not significantly decrease the viability of the cells, as measured by PrestoBlue fluorescence. In line with this, we could also still visualize phalloidin-labeled neurons 24 h after SNAP (Figure 2E).

### SNAP Outperforms Alternative Labeling Strategies

**Figure 3:**
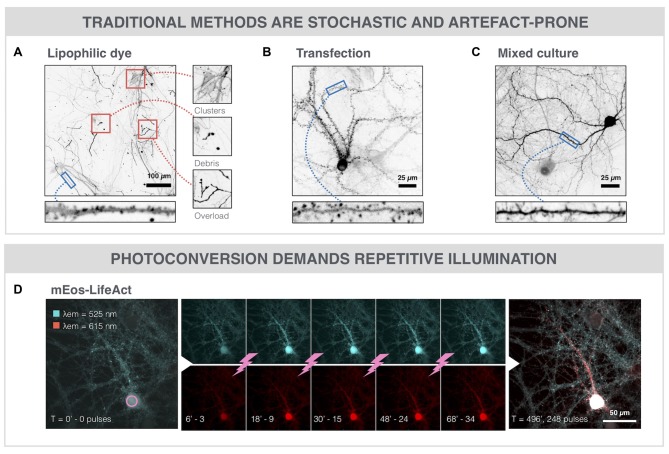
SNAP outperforms alternative labeling strategies. **(A)** The lipophilic dye DiI enables sparse, but stochastic labeling of neurons within the neuronal network. It suffers from drawbacks such as clustered staining of adjacent neurons, debris, and uneven dye loading or overloading. **(B)** Microscopic image of a neuron transduced with actin-RFP (BacMam vector). Spines can clearly be resolved since actin is highly enriched in spines. However, the overexpression of actin-RFP may lead to artifacts such as enlarged spine heads (see also Supplementary Figure S1B) or increased cellular stress. **(C)** Microscopic image of a Thy1-YFP neuron in a mixed culture with non-labeled WT cells (1:5 ratio). Spines can be discerned, but the signal of YFP in spines is weak and the labeled cell distribution is stochastic. **(D)** Photoconversion of mEos-LifeAct (cyan) in a primary neuron. Every 2 min, a 405 nm photoconversion pulse is delivered to the soma of the selected neuron, leading to a gradual spreading of the photoconverted (red) variant throughout the cytoplasm.

To expose the advantages of SNAP, we compared it with existing spine labeling strategies. The current standard for spine labeling is the use of the lipophilic dye DiI for membrane staining (Figure 3A). Although the intense labeling allowed spine identification in selected regions, the images needed to be manually scrutinized to find the dendrite stretches that could be unambiguously analyzed. In a randomly acquired image dataset, less than 5% (3.2 ± 2.8%) of the actual stained surface area was amenable to spine analysis (Supplementary Figure S1A). Only a low efficiency was reached because DiI labeling is inherently stochastic and was associated with a number of artifacts, such as debris, membrane fragments and uneven fluorescence intensity, as well as labeling of non-neuronal cells (e.g., astrocytes). An alternative method that we tested to label spines in dense cultures is based on a genetically encoded fusion protein actin-RFP (BacMam actin-RFP). We tuned the transduction protocol so as to obtain low efficiency, yielding only a few dozen labeled neurons in the network with high expression levels (Figure 3B). Dendritic spines could clearly be resolved, but again, lack of spatial control resulted in labeling of adjacent neurons (clusters) and variability in labeling efficiency across replicates. Moreover, spine head diameters were increased compared to other labeling protocols (Supplementary Figure S1B), pointing to overexpression artifacts. To avoid these enlarged spine heads, we tested a third approach by mixing a culture of genetically labeled cells with non-labeled cells. This was achieved by using Thy1-YFP mouse-derived neurons (YFP+), which constitutively produce YFP throughout the cell body. When mixing these YFP+ neurons with neurons from a WT Black6 mouse at a 1:5 ratio, sufficiently sparse cultures were obtained to resolve individual spines (Figure 3C). Although these cells integrated both morphologically and functionally in the network (Supplementary Figures S2A–C), the rather limited YFP intensity in spines and the stochastic, sometimes clustered, distribution of YFP+ neurons in the well, render this method suboptimal for automated spine analysis. Furthermore, in aged (21 DIV) cultures we found that the YFP signal accumulated in all neurons. This increased the background signal and hampered spine identification further, especially at later time points (Supplementary Figure S2D). A crucial drawback of the aforementioned strategies remains the lack of spatiotemporal control. Therefore, we also explored the potential of a targeted approach based on photoconversion of an actin-binding fluorescent protein (Figure 3D). AAV-mediated expression of mEos-LifeAct resulted in green fluorescent signal in all neurons of the network. Four-hundred and five nanometer photoconversion pulses were directed to the soma of a selected neuron, eventually leading to a bright red fluorescent signal in proximal as well as distal neurites of the targeted neuron. However, repeated photoconversion pulses (1 pulse every 2 min for 8 h) were needed to convert a sufficiently large amount of mEOS-LifeAct proteins to fill up the very extremities (*i.e.*, spines) of the neuron. Thus, next to the fact that this is a slow procedure, it puts a high energy load onto the cell which increases the risk on inducing phototoxicity. In contrast, the SNAP procedure requires a single nanosecond laser pulse per cell to permeabilize its membrane, allowing hundreds of cells to be labeled in one run, followed by a 1 h incubation period to allow intracellular diffusion of the phalloidin (Figure 1A). Owing to its high selectivity, SNAP raises the labeling efficiency of analyzable dendrite stretches to 25% (24.6 ± 9.4%) as compared to the gold standard (DiI labeling; Supplementary Figure S1A), whilst retaining the morphological integrity of spines (Supplementary Figure S1B).

### SNAP Reveals Maturation-Dependent and Hypoxia-Induced Changes in Spine Density

**Figure 4:**
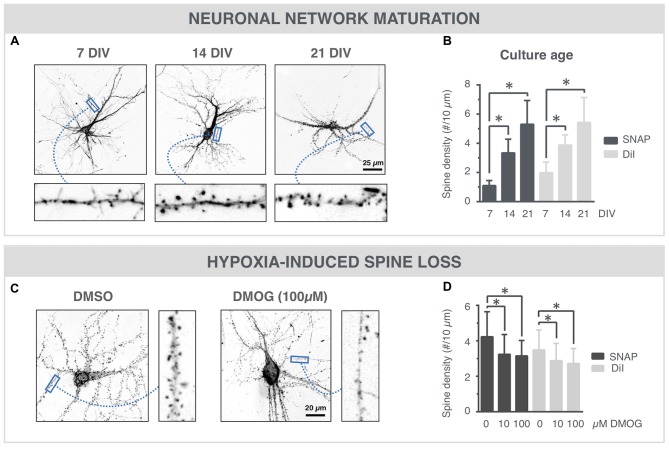
SNAP reveals maturation-dependent and hypoxia-induced changes in spine density. **(A)** Microscopic images of phalloidin-labeled neurons at 7, 14 and 21 DIV. Spines (arrows) can be discerned at different time points. **(B)** Quantification shows a time-dependent increase in spine density and a similar trend for SNAP and DiI labeling (*p < 0.05 in Dunn all pairs test, see Table 1 for *p*-values). **(C)** Phalloidin-labeled neurons with and without DMOG treatment to mimic hypoxic conditions. **(D)** Quantification of spine density after SNAP or DiI labeling shows a reduction in spine density upon 24 h DMOG treatment (*p < 0.05 in Steel test, see Table 1 for *p*-values). Microscopy images have been inverted for clarity.

Spine density is often used as a quantitative readout for synaptic connectivity within the neuronal network (Papa et al., 1995; Verstraelen et al., 2014). To further validate the SNAP approach, we compared its performance with the standard, but stochastic and artifact-prone lipophilic dye (DiI) labeling. The high-resolution images of Figure 4A show SNAP phalloidin-labeled neurons at 7, 14 and 21 DIV, in which spines could clearly be resolved. After quantification, an age-dependent increase in spine density was detected (Figure 4B), with a strong commonality between SNAP and DiI labeling (Pearson’s correlation 0.67). DMOG, an inhibitor or prolyl-4-hydroxylase and inducer of hypoxia-inducible factor, has previously been shown to induce spine reduction (Segura et al., 2016). Figure 4C shows phalloidin-labeled neurons in the presence and absence of DMOG (24 h, 14 DIV). Spine density was quantified on SNAP- as well as DiI-labeled cultures, treated with 0, 10 or 100 μM DMOG for 24 h (Figure 4D). In both cases, our quantifications revealed a significantly reduced spine density after DMOG treatment.

## Discussion

With our work, we have demonstrated the potential of a novel intracellular delivery method based on AuNP-sensitized photoporation to selectively label primary neurons and their spines in dense neuronal networks. To visualize dendritic spines, the fluorescently functionalized actin-binding dye phalloidin was introduced in living neurons. This was done in a spatially resolved manner, after automatic selection of neurons that were sparsely distributed throughout the network.

The key concept behind SNAP is the use of sensitizing nanoparticles. Even though it was shown before that a precisely focused beam of highly intense light could permeabilize the plasma membrane with subcellular resolution (Tirlapur and König, 2002; Barrett et al., 2006), the use of AuNPs allows reducing the laser fluence (energy per surface area) so that the entire neuronal soma can be irradiated at once with a broad laser beam, thereby significantly increasing throughput. It was previously shown that the use of sensitizing AuNPs allows reducing the laser fluence by a factor of 400 and the total energy load per cell by 2.4 times, as compared to photoporation with a focused laser beam (Barrett et al., 2006; Xiong et al., 2014). In line with this, when using a single laser light pulse at a fluence of 2 J/cm^2^, we found SNAP to be non-toxic, at least within a 24-h time frame. Since this pulse was sufficient to load cells with phalloidin, we conclude that under these conditions the conversion of light into mechanical energy by the AuNPs is highly efficient and remains local (Xiong et al., 2016b). Thus, SNAP can effectively be used to assess spine density in a physiological manner.

Although it is not the prime purpose, we have successfully acquired images of neurons 24 h after SNAP labeling. However, it should be noted that phalloidin stabilizes actin filaments (Cooper, 1987) and may therefore alter the properties of the spines. Given its inability to cross the plasma membrane, phalloidin was rarely used in living cells. Yet, one report documents alterations in the growth and migration of epithelial cells and fibroblasts after micro-injection of phalloidin (Wehland et al., 1977). However, these authors have used much higher phalloidin concentrations (0.2 and 1 mM in the microinjection solution) compared to our study (0.7 μM in the extracellular medium). Although the latter may explain why we could still image apparently healthy neurons after 24 h, it is advisable to use other labels that interact with actin in a less stringent manner (e.g., fluorescently labeled LifeAct) or inert dyes such as fluorescently-labeled dextrans for long-term imaging (albeit at the expense of spine labeling quality, *cfr*. Thy1-YFP).

In assessing the performance of SNAP for spine labeling, we have made a comparison with other labeling strategies. A summary of their properties (targeting potential, labeling speed, spine signal to local background intensity ratio) and drawbacks is listed in Table 2. The current gold standard for spine labeling consists in applying hydrophobic dyes such as DiI via bath or ballistic application (Cheng et al., 2014). The latter makes use of a “gene gun” to shoot DiI-coated bullets into thick (e.g., 150 μm) tissue slices, but is less commonly used for labeling of 2D cultures (Staffend and Meisel, 2011). DiI labeling is associated with several staining artifacts, such as uneven dye loading, clustered cells and lack of cell type specificity, that preclude automated image analysis. Genetic labeling is a frequently used alternative approach, but overexpression of fusion proteins always comes with the concern of inducing artifacts, as also we noticed by the increased spine head size after actin-RFP overexpression. This artifact was not observed after mEos-LifeAct expression, since the fluorescent protein (mEos) was targeted to endogenous actin via the LifeAct sequence (Riedl et al., 2008), rather than expressing additional actin on top of the endogenous levels. Also, in Thy1-YFP neurons, spine heads were not enlarged, since the fluorescent protein does not specifically accumulate in spines but moves freely in the cytoplasm. However, the background YFP signal increased markedly over time, making reliable spine analysis difficult. We have seen an increase in background fluorescence in the vast majority of neurons in mixed culture, although only one out of five was transgenic. This signal increase was not visible in aged WT (non-mixed) control cultures. Since the neurons do not divide, our observations suggest that cytosolic YFP was transferred from transgenic to non-transgenic neurons in the mixed culture. Although to our knowledge, YFP transfer was not yet documented in literature, inter-neuronal protein transfer has been amply shown for α-synuclein (Desplats et al., 2009), β-amyloid (Song et al., 2014) and Tau (Wang et al., 2017). Potential mechanisms that were shown to be involved include synaptic transfer (Dujardin et al., 2014), exosomal exchange (Chivet et al., 2014) and transport through tunneling nanotubes (Tardivel et al., 2016). A detailed analysis of the presumed YFP transfer was beyond the scope of this study, and would require quantification of the fraction of YFP+ neurons in mixed cultures with a neuronal counterstain, or follow-up of transmission between YFP+ and YFP− cells using microfluidic microchannels (Dujardin et al., 2014).

**Table 2:** Comparison of sparse spine labeling strategies.

Sparse labeling approach	Targeting	Speed	Spine SBR	Ease-of-use	Artifacts and disadvantages
Lipophilic dye (DiI)	−	+	±	+	Debris, uneven dye loading, clusters, no cell type specificity
Transfection (actin-RFP)	−	−	+	+	Increased spine head size, clusters
Mixed culture (YFP)	−	−	−	−	Clusters, low flexibility
Photoconversion (mEos-LifeAct)	+	−	+	−	Repeated pulses needed, risk of photodamage
SNAP (AF488-phalloidin)	+	+	+	±	Pulsed laser needed

*Commonly used spine labeling strategies are compared with SNAP in terms of targeting potential, labeling speed, spine SBR (average spine signal to local background intensity ratio), applicability and risk for artifacts*.

In non-targeted labeling approaches, the stochastic and sometimes clustered distribution of cells throughout the well complicates automated spine analysis. Targeted labeling strategies are, therefore, highly desirable. Micro-injection in selected cells has been successfully used, but this approach lacks the speed of the SNAP method (Dumitriu et al., 2011; Chow et al., 2016). As an alternative for SNAP, we explored the potential of an actin-binding photoconvertible protein, but found that repeated 405 nm laser pulses were needed to reach sufficiently high levels of the photoconverted variant. In case of SNAP, a single light pulse was sufficient to label a selected neuron. Therefore, the throughput for labeling many neurons was significantly higher compared to the photoconversion approach (especially when using the automated image-guided mode). As a proof-of-concept for SNAP-mediated spine analysis, maturation-dependent spine density was investigated, which increased over time in line with previous reports (Papa et al., 1995). Chemical induction of hypoxic stress led to a decreased spine density, in accordance with a study where spines were visualized by overexpression of cytosolic fluorescent proteins (Segura et al., 2016). Although we have now solely focused on measuring changes in spine density, the analysis could easily be extended to the analysis of spine morphology. Given the small nature of spines, such analysis could however benefit from a combination of SNAP with super-resolution imaging. Yet, the relevance of spine morphology measurements is still unclear since the exact relation between spine morphology and synaptic strength is the subject of ongoing debate (Tønnesen et al., 2014; Segal, 2017).

Though the current study involves the selective labeling of actin and hence dendritic spines with phalloidin, SNAP’s range of potential applications is much wider. First, the selective labeling can be directed to specific neurons that show particular geno- or phenotypic patterns of interest. These may be other cell types such as astrocytes, neuronal subtypes (e.g., pyramidal- or interneurons) or neurons that show intracellular accumulation of toxic proteins (e.g., α-Synuclein or Tau). Second, consecutive labeling rounds with different fluorochromes allows studying the connectivity pattern between neurons of different subpopulations. Proof for the feasibility of both of these applications was provided in this study (Figure 2). Third, in addition to labeling selected cells with impermeable fluorescent dyes, it may be equally possible to selectively deliver other nanomaterials such as drugs or siRNA, as previously shown in cell lines (Lukianova-Hleb et al., 2012; Xiong et al., 2014). Lastly, SNAP bears the potential to label cells in a 3D environment such as organotypic brain cultures. However, it remains to be determined whether AuNPs penetrate well in brain tissue, and precise axial focusing of the photoporation beam will be needed to achieve single cell selectivity.

In conclusion, the flexibility and speed with which individual cells can be labeled make SNAP an ideal tool for high-content applications, not only for selective labeling but also for targeted drug or nucleic acid delivery. This may eventually lead to a better understanding of the pathogenic mechanisms that underlie neurodevelopmental and neurodegenerative disorders.

## Author Contributions

RX, PV, KB, WHV conceived and designed the experiments, and drafted the manuscript. RX and PV performed the experiments. RX, PV, JD, AGS, J-PT, SCS, WHV and KB critically revised the manuscript. All authors are accountable for all aspects of the work.

## Conflict of Interest Statement

The authors declare that the research was conducted in the absence of any commercial or financial relationships that could be construed as a potential conflict of interest.
