# Supplementary material for: Selective Labeling of Individual Neurons in Dense Cultured Networks With Nanoparticle-Enhanced Photoporation
Source: Front Cell Neurosci. 2018 Mar 29;12:80. doi: 10.3389/fncel.2018.00080 (PMC5884872; doi:10.3389/fncel.2018.00080)
Supplement: Supplementary file 1 [file Data_Sheet_1.pdf]

Supplementary Material

Selective Labeling of Individual Neurons in Dense Cultured Networks with Nanoparticle-enhanced Photoporation

Ranhua Xiong, Peter Verstraelen, Jo Demeester, Andre G. Skirtach, Jean-Pierre Timmermans, Stefaan C. De Smedt, Winnok H. De Vos and Kevin Braeckmans

Supplementary materials and methods

Quantification of spine head diameter and analyzable dendrite fraction

Spine head diameters were measured manually in ImageJ software by randomly selecting 10 spines per image. The data was averaged per image and 15 images originating from 3 isolations were considered for each labeling method. The labeling specificity was estimated on 14 DIV cultures after DiI and SNAP labeling (10 images each). The DiI images were acquired at random positions, while the phalloidin images were acquired at the positions determined during image-guided SNAP. The area percentage of analyzable dendrites was determined in ImageJ. First, the area of all stained structures was measured using Huang thresholding. Next, analyzable dendrite stretches, *i.e.* isolated stretches of at least 50 µm, were manually selected and measured. In case multiple analyzable stretches were present, their area was added together before calculating the analyzable area fraction.

Characterization of mixed cultures

For the determination of dendritic spine density in mixed WT/YFP cultures, 14 DIV cultures were fixed (2% paraformaldehyde in 0.1 M phosphate buffer, 20 min at RT) and stained with the far-red DiI variant DiD (2 µM in PBS, 20 min, ThermoFisher V22887). Spine density was determined on YFP-negative and -positive neurons from within the same cultures (n=3 isolations, for each isolation >1000 µm of dendrite was analyzed per condition). To assess functional integration, WT and mixed cultures of 7 and 14 DIV were subjected to calcium imaging with a red-shifted calcium indicator (GFP-certified Fluoforte, Enzo Life Sciences ENZ-52016-5C50). Live cell imaging and image analysis were carried out as described before (Cornelissen et al., 2013). To quantify the synchronicity of spontaneous calcium bursts, the average Pearson's correlation coefficient across neurons within the field-of-view was calculated. The data originated from 3 isolations with 3 wells per isolation per condition.

Supplementary figures

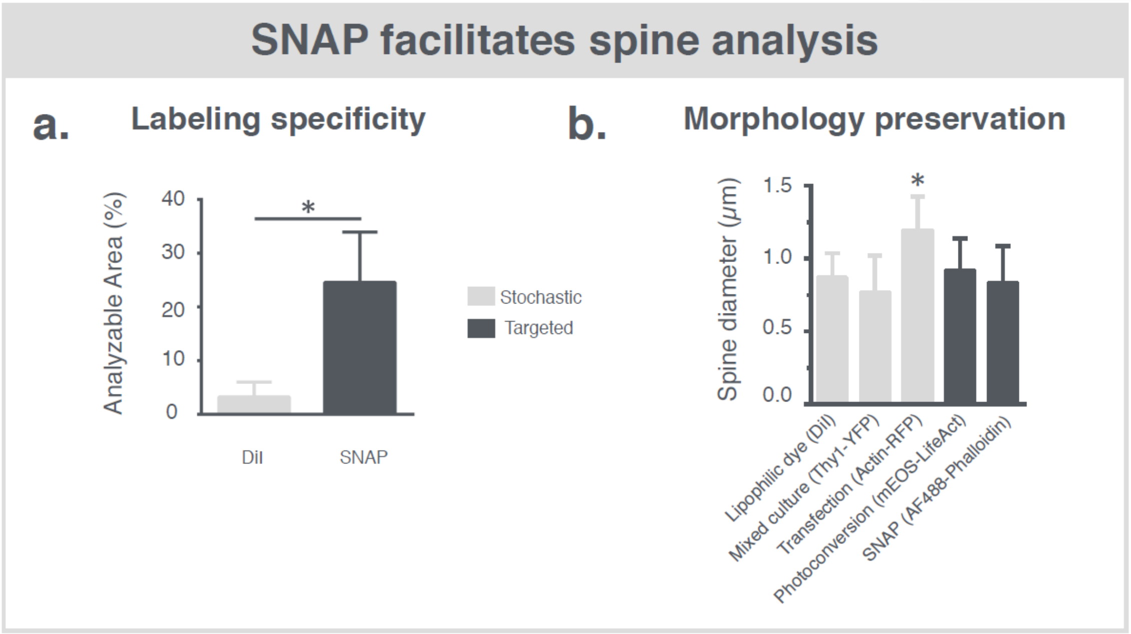

Supplementary Figure 1 - SNAP facilitates spine analysis

a. Comparison of labeling specificity between DiI and SNAP in 14 DIV neurons, measured as percentage of the surface area of analyzable dendrite stretches divided by the total labeled surface area. **b.** Quantification of the spine head diameter shows an increased size of BacMam actin-RFP transfected spines, indicative of an overexpression artefact (*$p<0.05$ in Dunn all pairs test, see Table 1 for p-values), as compared to alternate spine labeling strategies, including SNAP.

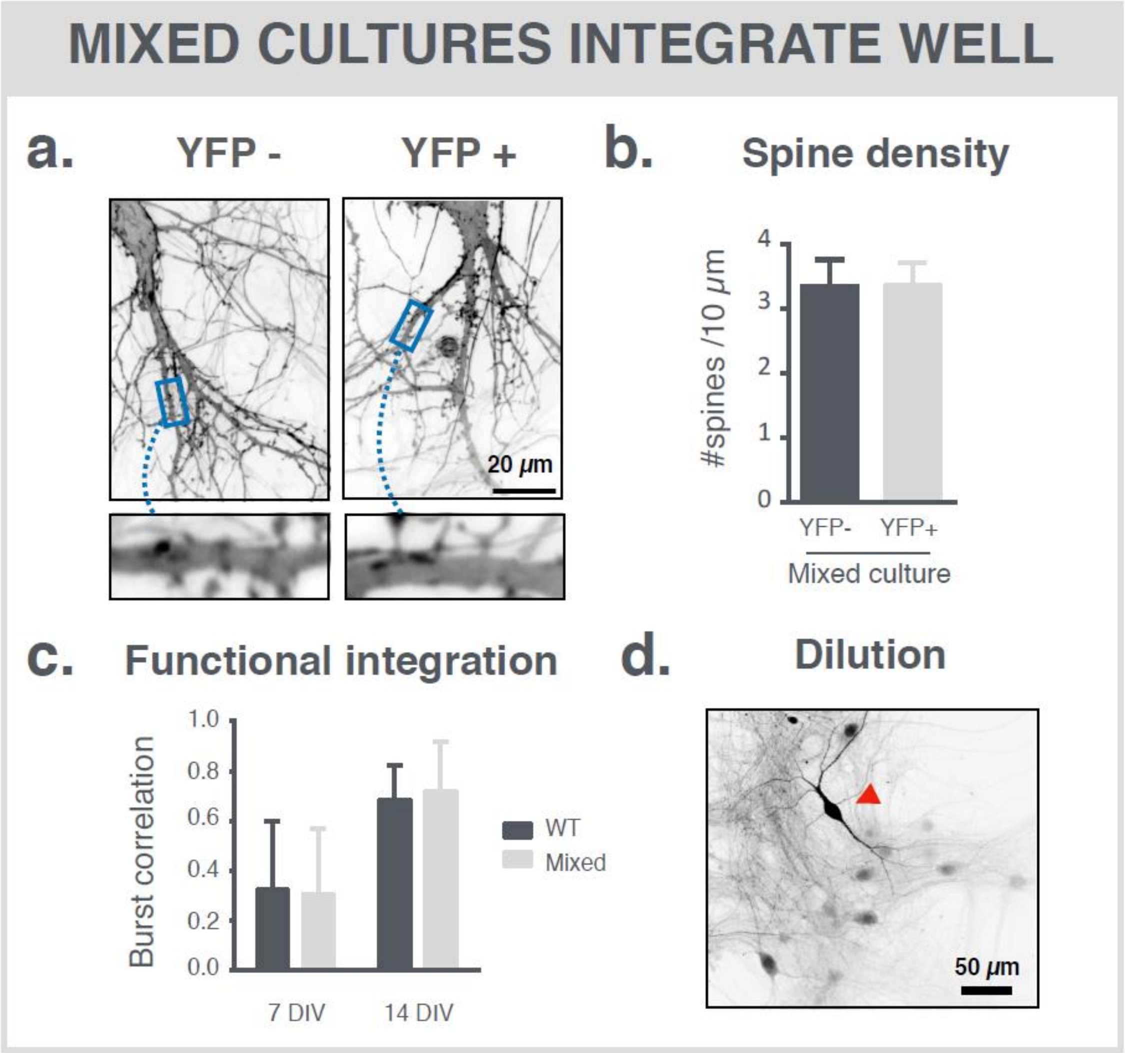

Supplementary Figure 2 - Characterization of mixed cultures
a. Representative microscopic images of a YFP-negative and -positive neuron in a mixed 14 DIV culture. Spines are visualized by lipophilic DiD staining. **b.** Spine quantification at 14 DIV reveals a similar density in WT and YFP neurons within the mixed cultures, indicative of the morphological integration of both neuronal populations. **c.** Live cell calcium imaging revealed a comparable burst correlation, *i.e.* the synchronicity of spontaneous calcium bursts across neurons, within WT and mixed cultures at 7 and 14 DIV (PCC: Pearson's correlation coefficient). **d.** Low-magnification image of a 21 DIV mixed culture, showing the problem of YFP dilution from transgenic (red arrowhead) to plausibly non-transgenic neurons. Microscopy images have been inverted for clarity.
